# Rational Design of the Platinahelicene Enantiomers for Deep-Red Circularly Polarized Organic Light-Emitting Diodes

**DOI:** 10.3389/fchem.2020.00501

**Published:** 2020-06-17

**Authors:** Zhi-Ping Yan, Xu-Feng Luo, Kang Liao, You-Xuan Zheng, Jing-Lin Zuo

**Affiliations:** State Key Laboratory of Coordination Chemistry, Jiangsu Key Laboratory of Advanced Organic Materials, School of Chemistry and Chemical Engineering, Nanjing University, Nanjing, China

**Keywords:** platinahelicene, circularly polarized light, thermal and configurational stability, CP-OLED, deep-red emission

## Abstract

[n]Helicene derivatives are most popular chiral structures to construct luminescent materials with circularly polarized (CP) light, which have revealed appealing application in chiral optoelectronics. Particularly, because of the unique phosphorescent emission, platinahelicene has great application prospects in CP organic light-emitting diode (CP-OLED). Herein, by decorating the pyridinyl-helicene ligand with trifluoromethyl (-CF_3_) unit in a specific position, a pair of platinahelicene enantiomers was prepared and separated with extremely twisted structure showing not only superior thermal and configurational stability but also good CP luminescence (CPL) property with dissymmetry factors (|*g*_PL_|) of 6 × 10^−3^. Moreover, the evaporated CP-OLEDs based on platinahelicene enantiomers exhibited the deep-red emission with the peak at 653 nm as well as obvious CP electroluminescence (CPEL) signals with the |*g*_EL_| in 10^−3^ order. Therefore, the design strategy provides an efficient way to improve the CPL properties of platinahelicene to cope with the future application in CP-OLEDs.

**Graphical Abstract:**
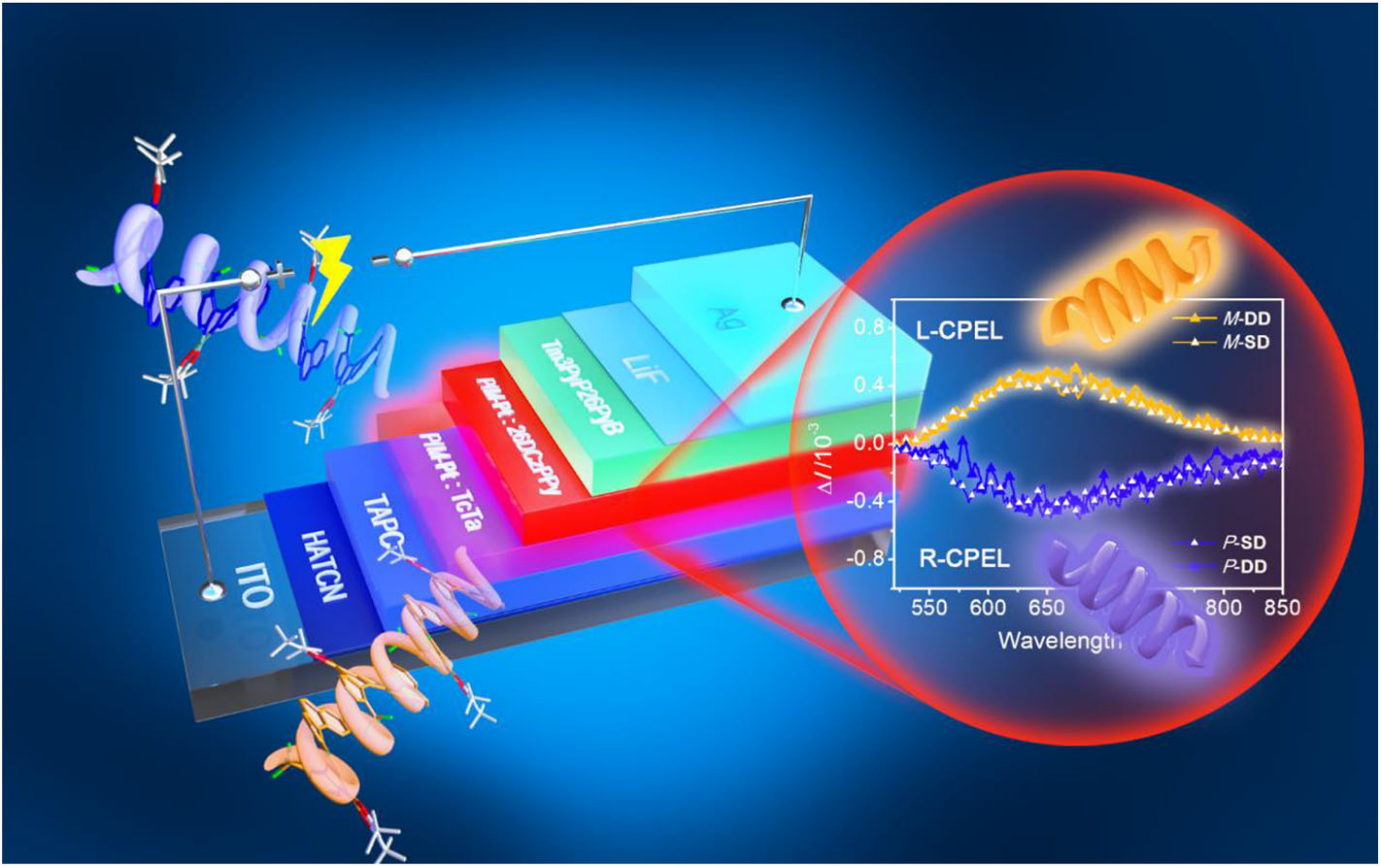
A pair of chiral deep-red platinahelicene enantiomers show good CPL property with |*g*_PL_| of 6.0 × 10^−3^. And the fabricated CP-OLEDs exhibit EQE_max_ of 4.6% with the |*g*_EL_| at the degree of 10^−3^.

## Introduction

In recent years, the exploration of chiral materials enabling circularly polarized (CP) light has attracted widespread attention in the field of chiral optoelectronics (Zhang et al., 2014). Compared with the traditional way of obtaining CP light (Grell et al., 2001) with polarizer, CP organic light-emitting diodes (CP-OLEDs) (Li et al., 2015, 2018) could emit CP light directly, which avoid brightness loss and complicated device structures. In term of current research (Han et al., 2018), it is still an important way to design chiral materials with excellent CPL properties for achieving high-performance CP-OLEDs. And the degree of circularly polarized photoluminescence (CPPL) or circularly polarized electroluminescence (CPEL) is characterized by the dissymmetry factor (*g*_PL_ or *g*_EL_) defined as *g* = 2 × Δ*I* /*I* =2 × (*I*_L_ - *I*_R_)/ (*I*_L_ + *I*_R_). For example, Meijer and co-workers (Peeters et al., 1997) firstly obtained CP electroluminescence (CPEL) by using a chiral-substituted poly(p-phenylenevinylene) derivative as emitter. Since then, there has been some progress in chiral luminescent materials and efficient CP-OLEDs. Notably, although several reported chiral lanthanide complexes (Zinna et al., 2015, 2017) show excellent CPL properties with the |*g*_PL_| values over 1, the poor device performances make them difficult to be applied for practical OLEDs. On the contrary, chiral transition metal complexes [Ir(III), Pt(II)] and thermally activated delayed fluorescence materials (Feuillastre et al., 2016; Han et al., 2017; Wu et al., 2019) exhibit increasingly satisfactory results in term of the electroluminescence properties. Nevertheless, most of these materials used in efficient OLEDs exhibit poor CPL properties with the |*g*_PL_*|* value in the range of 10^−5^ to 10^−3^ (Han et al., 2018; Yan et al., 2019b) and mostly <5 × 10^−3^. Hence, it is important to provide a design strategy to improve their CPL properties.

Generally, introducing chiral units to luminescent materials is an efficient way to construct CPL materials (Sakai et al., 2015; Sánchez-Carnerero et al., 2015). Compared with chiral carbon centers, the special conjugated screw-shaped structure of [n]helicene derivatives makes it possess intrinsic chirality, which is one of the most effective chiral units (Dhbaibi et al., 2019; Zhao et al., 2019). Particularly, platinahelicene (Norel et al., 2010; Hellou et al., 2017) has not only the chirality of helicene but also the properties of phosphorescent emission, making it a potential choice for efficient CP-OLEDs. For example, Crassous and co-workers ((Norel et al., 2010), (Shen et al., 2014)) developed a series of chiral platinahelicene exhibiting both phosphorescent and CP emission. Their elegant molecular design was based on the introduction of pyridinyl-helicene ligand into cyclometalated complexes. Later, a solution processed CP-OLED based on platinahelicene (Brandt et al., 2016) was reported by Fuchter group, achieving a very high |*g*_EL_| value of 0.38 with a maximum luminance (*L*_max_) of 222 cd m^−2^ and a maximum current efficiency (η_max_) of 0.25 cd A^−1^, indicating the application of platinahelicene in CP-OLEDs. However, owing to the poor volatility, this kind of platinahelicene is difficult to be fabricated by vacuum deposition to gain better device performances. Subsequently, we modified the platinahelicene with the sterically hindered -CF_3_ and -^t^Bu group to enable the material to be sublimated for the preparation of evaporated devices (Yan et al., 2019a), showing the |*g*_EL_| of (1.1–1.6) × 10^−3^ with a *L*_max_ of 11,590 cd m^−2^ and a η_max_ of 22.52 cd A^−1^, respectively. Meanwhile, the device performances are the highest values among the reported devices based on chiral phosphorescent Pt(II) complexes. Although the device performances were improved a lot, the |*g*_PL_| of 3.7 × 10^−3^ still need to be promoted.

Herein, we created helicene core through a special structure design combining the -CF_3_ group and helicene structure, and further improving the CPL property of platinahelicene. Compared with the platinahelicene reported by Crassous and Fuchter (Norel et al., 2010; Brandt et al., 2016), (*RAC*)-**Pt** possesses superior thermal stability and could be easily sublimated owing to the introduction of additional large hindered groups (-CF_3_, ^t^Bu). Meanwhile, the special substitution position of the group not only makes the helicene structure more configurationally stable, but also endows this complex better CPL performance because of the more twisted structures. Therefore, based on our design strategy we successfully prepared a new kind of platinahelicene which was further prepared evaporated CP-OLED with good performance. In this way, *P*-**Pt** and *M*-**Pt** separated by (*RAC*)-**Pt** show higher *g*_PL_ of −5.9 × 10^−3^/6.0 × 10^−3^. And both deep-red electroluminescence (λ_max_ = 653 nm) and CP light activity were achieved by fabricating the evaporated OLEDs.

## Materials and Methods

### General Method

NMR measurements were conducted on a Bruker AM 400 spectrometer. The mass spectra were recorded by an electrospray ionization (ESI) mass spectrometer (LCQ fleet, Thermo Fisher Scientific) and Matrix Assisted Laser Desorption Ionization Time of Flight Mass Spectrometry (autoflex TOF/TOF, Bruker Daltonics), high-resolution mass spectra were recorded on a MICROTOF-Q III instrument. Ultraviolet-visible (UV-vis) absorption spectra were measured on a UV-3100 spectrophotometer and PL spectra were obtained from a Hitachi F-4600 photoluminescence spectrophotometer. The absolute photoluminescence quantum yields (Φ) and the decay lifetimes of the complex were measured by HORIBA FL-3 fluorescence spectrometer. Thermogravimetric analysis (TGA) was performed on a Pyris 1 DSC under nitrogen at a heating rate of 10°C min^−1^. (*RAC*)-**Pt** was separated by CHIRALPAK IE (IE00CD-RH008) column which was employed as stationary phase and the hexane/dichloromethane (80/20) was employed as eluent. Cyclic-voltammetry measurement system carried out at room temperature in deaerated CH_3_CN, employing a polished Pt plate as the working electrode, terta-n-butylammonium perchlorate (0.1 M) as the supporting electrolyte and Fc^+^/Fc used as the reference, with the scan rate of 0.1 V/s. The HOMO and LUMO levels were obtained by the following equation: *E*_HOMO_ = –(4.8+*E*_oxonset_) (eV), *E*_LUMO_ = –(*E*_HOMO_+*E*_g_) (eV), and *E*_g_ was estimated from the UV-vis absorption spectra, *E*_g_ = *hc*/λ_abs_. The circular dichroism (CD) and CPL spectra were measured in the same condition with UV-Vis absorption spectra and PL spectra. The CD spectra were measured on a Jasco J-810 circular dichroism spectrometer with “low” sensitivity. The circularly polarized photoluminescence (CPPL) and circularly polarized electroluminescence (CPEL) spectra were measured on a Jasco CPL-300 spectrophotometer based on “Continuous” scanning mode at 200 nm/min scan speed. The test mode adopts “Slit” mode with the *E*_x_ and *E*_m_ Slit width 3,000 μm and the digital integration time (D.I.T.) is 2.0 s with multiple accumulations (10 times or more).

### Theoretical Calculation

All the DFT and TD-DFT calculations were carried out using Gaussian 16 software package. The initial structures were created according to the GaussView 6 based on crystal structures. The ground state geometry optimization with frequent calculations for all the complexes were performed using B3LYP exchange-correlation functional. On the basis of the optimized structures, vertical transition energy calculations were carried out with B3LYP functional, too. For all the calculations, a combination of basis sets that Lanl2dz for platinum and 6-31G (d,p) for the others were employed and the solvent effect were considered by C-PCM model in CH_2_Cl_2_.

### Device Fabrication

All the final products were purified by sublimation before applied to device fabrication. Moreover, the OLEDs with the emission area of 0.1 cm^2^ were fabricated on the pre-patterned ITO-coated glass substrate with a sheet resistance of 15 Ω sq^−1^. The ITO glass substrates were cleaned with ITO lotion and deionized water and then dried in an oven at 120 °C. The devices were fabricated by vacuum deposition of the materials at <10^−4^ Pa with the deposition rate of 1–2 Å s^−1^. The platinahelicene and host were co-evaporated from two separate sources. The LiF and Al were deposited with the rate of 0.1 and 3 Å s^−1^, respectively.

## Result and Discussion

### Preparation and Characterization of Platinahelicene

**Scheme 1:**
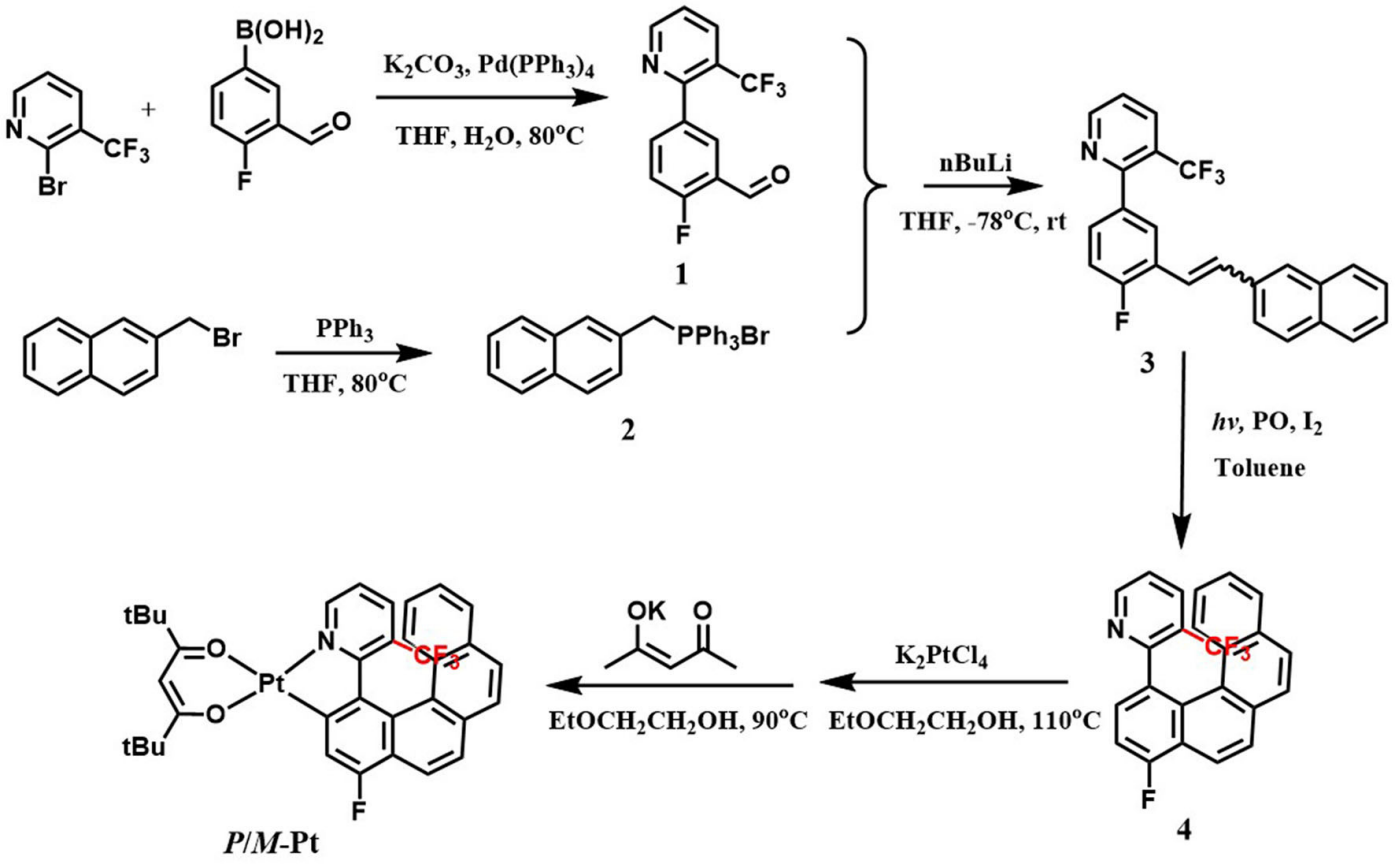
Synthetic routes and molecular structure of (*RAC*)-**Pt**.

All experiments were performed under nitrogen atmosphere and the relevant procedures referred to the reported literature (Norel et al., 2010). The starting reactants and solvents were used as commercial grade without further purification. The synthetic routes of (*RAC*)-**Pt** are depicted in Scheme 1. The helicene ligand **4** and (*C*^*N*^)Pt(μ-Cl) chloride-bridged dimmer were prepared by the reported methods. Then the platinahelicene was readily obtained by mixing the μ-chloro-bridged dimer with 2,2,6,6-tetramethylheptane-3,5-dione sodium salt. The target platinahelicene was purified by column chromatography and sublimation. Due to the rapid racemization at room temperature, the helicene ligand is difficult to be separated into stable enantiomers. On the contrary, with the introduction of coordination bond, *P*-**Pt** and *M*-**Pt** are configurationally stable, which could be separated by (*RAC*)-**Pt** by using chiral HPLC with enantiomeric purity higher than 99%.

### 1-Fluoro-4-(6-Trifluoromethyl-2-Pyridyl)-Benzo[*g*]Phenanthrene (4)

The mixture of *cis* and *trans* **3** (1.0 g, 2.55 mmol), *I*_2_ (30 mg, 0.12 mmol) and 10 ml propylene oxide were dissolved in toluene (1,000 mL). The solution was irradiated for 4 h by 250 W mercury vapor lamps. The solvent was evaporated under reduced pressure and then the residue was purified by column chromatography [silica gel, ethyl acetate: petroleum ether 1:10 (v/v)] to afford **4** as a beige solid (598 mg, 60%). ESI-MS, calculated: m/z 392.11 for [M+H]^+^ (C_24_H_14_F_4_N)^+^ found: m/z 392.25. ^1^H NMR (400 MHz, CDCl_3_): δ 9.05 (d, *J* = 3.7 Hz, 1H), 8.27 (d, *J* = 8.6 Hz, 1H), 8.03 (dd, *J* = 19.2, 8.1 Hz, 1H), 7.92 (dt, *J* = 8.4, 4.3 Hz, 2H), 7.89-7.77 (m, 3H), 7.72 (d, *J* = 8.0 Hz, 1H), 7.44 (dt, *J* = 31.7, 8.8 Hz, 1H), 7.30 (d, *J* = 7.8 Hz, 1H), 7.22 (t, *J* = 7.4 Hz, 1H), 6.99 (t, *J* = 7.3 Hz, 1H).

### (6-Trifluoromethyl-2-[4′-(1′-fluoro-benzo[g]phenanthrenyl)]pyridinato-*N, C*)platinum-*tert*-butylacetylacetonate (*RAC*)-Pt

**1) Preparation of the platinum μ-chloro-bridged dimer:** To a solution of **4** (400 mg, 1.02 mmol) in ethoxyethanol (30 mL) was added K_2_PtCl_4_ (423 mg, 1.02 mmol). The suspension was gently warmed with stirring until all the platinum salt dissolved. The solution was then refluxed for 16 h to yield a dark green suspension. After cooled to room temperature, water (100 mL) was added. The precipitation was filtered and dried in air and then dark yellow solid (549 mg, 88%) was obtained.

**2) Preparation of (*RAC*)-Pt:** To a solution of μ-chloro-bridged dimer (549 mg, 0.44 mmol) in ethoxyethanol (25 mL) was added the 2,2,6,6-tetramethylheptane-3,5-dione sodium salt (217 mg, 0.97 mmol). The reaction mixture was refluxed overnight and then concentrated under reduced pressure. Purification by column chromatography (silica gel, ethyl acetate: petroleum ether 1:4 (v/v)), yielded (*RAC*)-**Pt** as orange-red solid (337 mg, 50%). MALDI-TOF-MS, calculated: 768.194 for M (C_35_H_31_F_4_NO_2_Pt), found: 768.382; HR-MS, calculated: 769.2011 for [M+H]^+^ (C_35_H_32_F_4_NO_2_Pt)^+^, found: 769.2013. ^1^H NMR (400 MHz, CDCl_3_): δ 9.25 (d, *J* = 4.6 Hz, 1H), 8.21 (d, *J* = 8.5 Hz, 1H), 8.11 (d, *J* = 8.5 Hz, 1H), 7.84 (q, *J* = 8.6 Hz, 2H), 7.79 (dd, *J* = 8.0, 5.1 Hz, 2H), 7.58 (d, *J* = 9.3 Hz, 1H), 7.34 (d, *J* = 6.9 Hz, 1H), 7.24 (t, *J* = 7.3 Hz, 1H), 7.05-6.99 (m, 1H), 6.92 (t, *J* = 7.7 Hz, 1H), 5.91 (s, 1H), 1.34 (d, *J* = 6.2 Hz, 18H).

**Figure 1:**
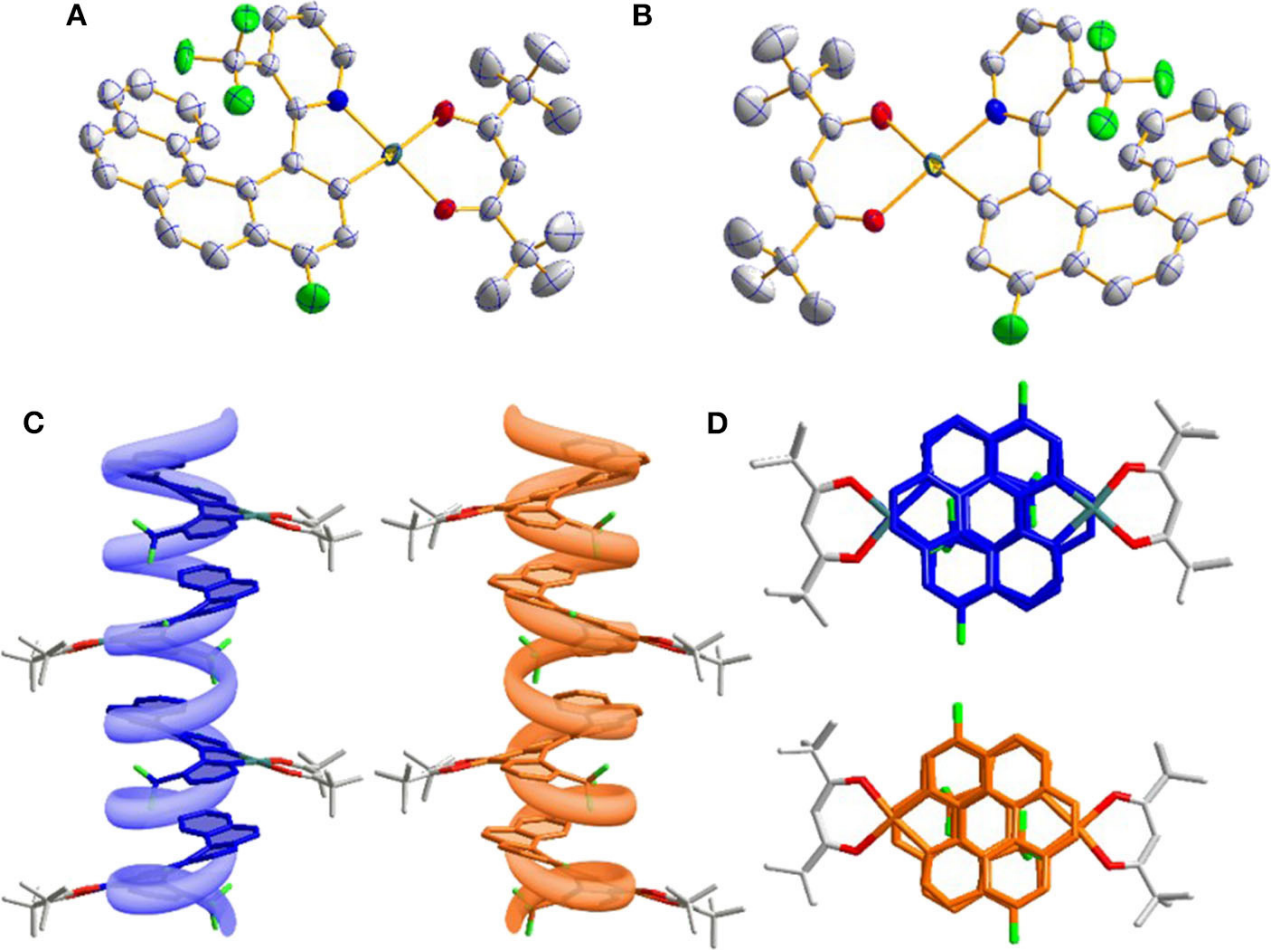
**(A)** Oak Ridge thermal ellipsoidal plot (ORTEP) diagrams of *P*-**Pt** (CCDC no. 1844425) and **(B)** *M*-**Pt** (CCDC no. 1844426); **(C)** molecular packing in crystal along b axis observed from side view and **(D)** top view.

The single crystals of two enantiomers were obtained by vacuum sublimation and the single crystal diffraction analysis further confirmed the preconceived structure. As shown in Figures 1A, **5B**, two symmetric enantiomers adopted twisted planar structures could be easily divided into *P* and *M* configurations. Interestingly, because the -CF_3_ group is inserted into the helicene-like skeleton, the twist angle of pyridine and the coordinate five-membered rings is 17.3°, bigger than the relevant report (Norel et al., 2010; Yan et al., 2019a), and the consecutive twist angles between the fused rings (10.6, 12.1, 12.3, and 9.9°) lead to a helical curvature (*hc*, the angle between the terminal helicene rings) value of 38.7° in *P*-**Pt** and *M*-**Pt**, which is smaller than the conventional organic or heteroatomic [6]helicene system (Graule et al., 2009). Although the helicene-based ligand shows a large distortion, the central Pt(II) still adopts a planar coordination form with the dihedral angle 5° between plane O_1_-Pt-O_2_ and N_1_-Pt-C_1_. Moreover, the smaller *hc* value leads to relative strong π-π interactions, as a result, *P*-**Pt** and *M*-**Pt** pile up in a parallel face-to-face style. As shown in Figures 1C,D, combining with the help of the special chiral *P* or *M* helicene unit, these strong interactions can self-assemble *P*-**Pt** and *M*-**Pt** to form *P* and *M* spiral structures, which may be a reason of the good CPL properties of the platinahelicene.

### Thermal and Configurational Stability

Thermal and configurational stability was investigated by TGA and CD spectra, respectively, which are vital parameters for chiral materials to fabricate CP-OLEDs. As shown in Figure S12, the decomposition temperature (*T*_d_, 5% loss of weight) of (*RAC*)-**Pt** is 321°C, which benefits the application during the operation of CP-OLEDs. Moreover, due to the introduction of large sterically hindered groups, these enantiomers are endowed with a better configurational stability, which could preserve the original configuration (Figure S11) even at 240^o^C under vacuum of 1 × 10^−4^ Pa overnight.

### Electrochemical Property and Theoretical Calculation

**Figure 2:**
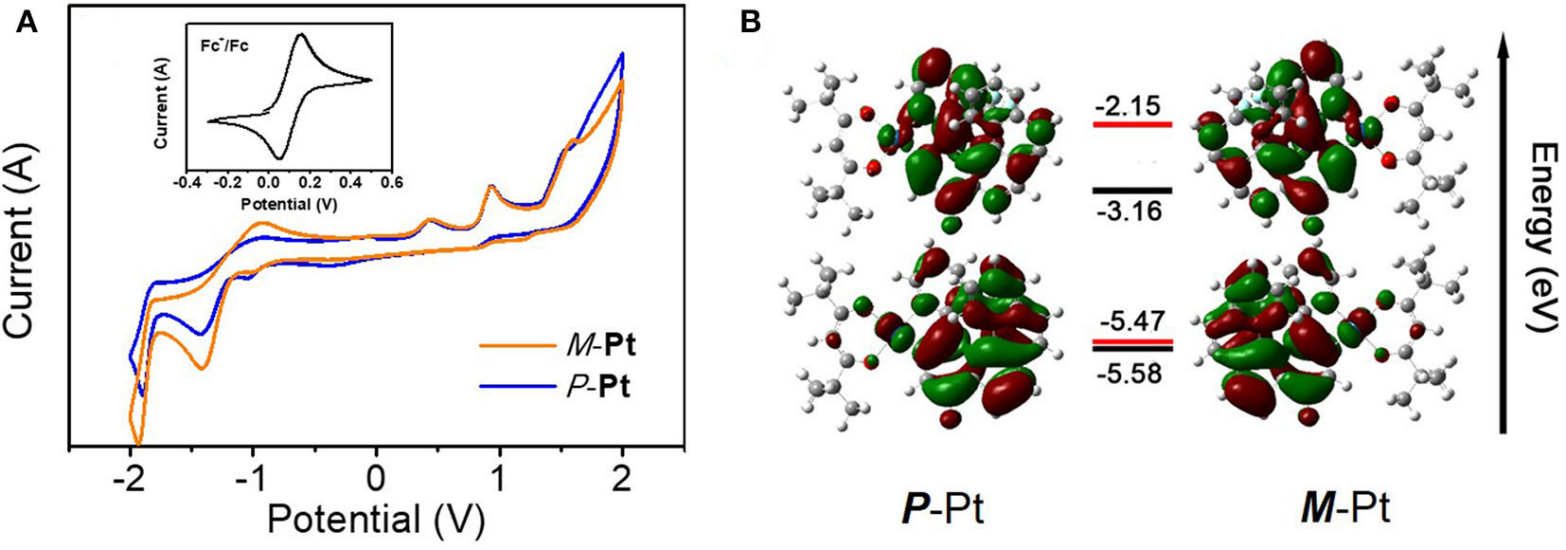
**(A)** The cyclic voltammogram curves and **(B)** HOMO/LUMO energy levels (experiment: black; calculation: red) and electronic cloud distributions of *P*-**Pt** and *M*-**Pt**.

Cyclic voltammetry (CV) measurements and density functional theory (DFT) calculations were further conducted to investigate the electronic and structural features of *P*-**Pt** and *M*-**Pt**. As depicted in Figure 2A, both complexes have irreversible oxidation and reduction waves and the electrochemical data are summarized in Table S3. The oxidation onsets of these two enantiomers reference Fc^+^/Fc are the same with the value of 0.78 V. Moreover, both *P*-**Pt** and *M*-**Pt** display the first reduction potential at about −1.43 V. These results demonstrated the chiral configuration has ignored influence on the electrochemical properties of the enantiomers. Therefore, the calculated highest occupied molecular orbital (HOMO) and the lowest unoccupied molecular orbital (LUMO) levels for *P*-**Pt** and *M*-**Pt** are −5.58/−3.24 eV, respectively, which are helpful for the carriers' injection and transport in OLEDs.

To gain a better insight of the electron cloud distributions and structure features, the molecular simulations were further executed by density functional theory (DFT) preformed with Gaussian 09 software, and the accurate energy and location of HOMO/LUMO were calculated by QMForge program. As shown in Figure 2B, the HOMO/LUMO are mainly distributed in the helicene ligand (77.09/91.51%) together with *d* orbitals of Pt atom (18.11/6.64%), respectively. Obviously, the distributions of HOMO/LUMO on ancillary ligand are very small, indicating the little influence on the energy level of this Pt(II) complex.

### Photophysical Property

**Figure 3:**
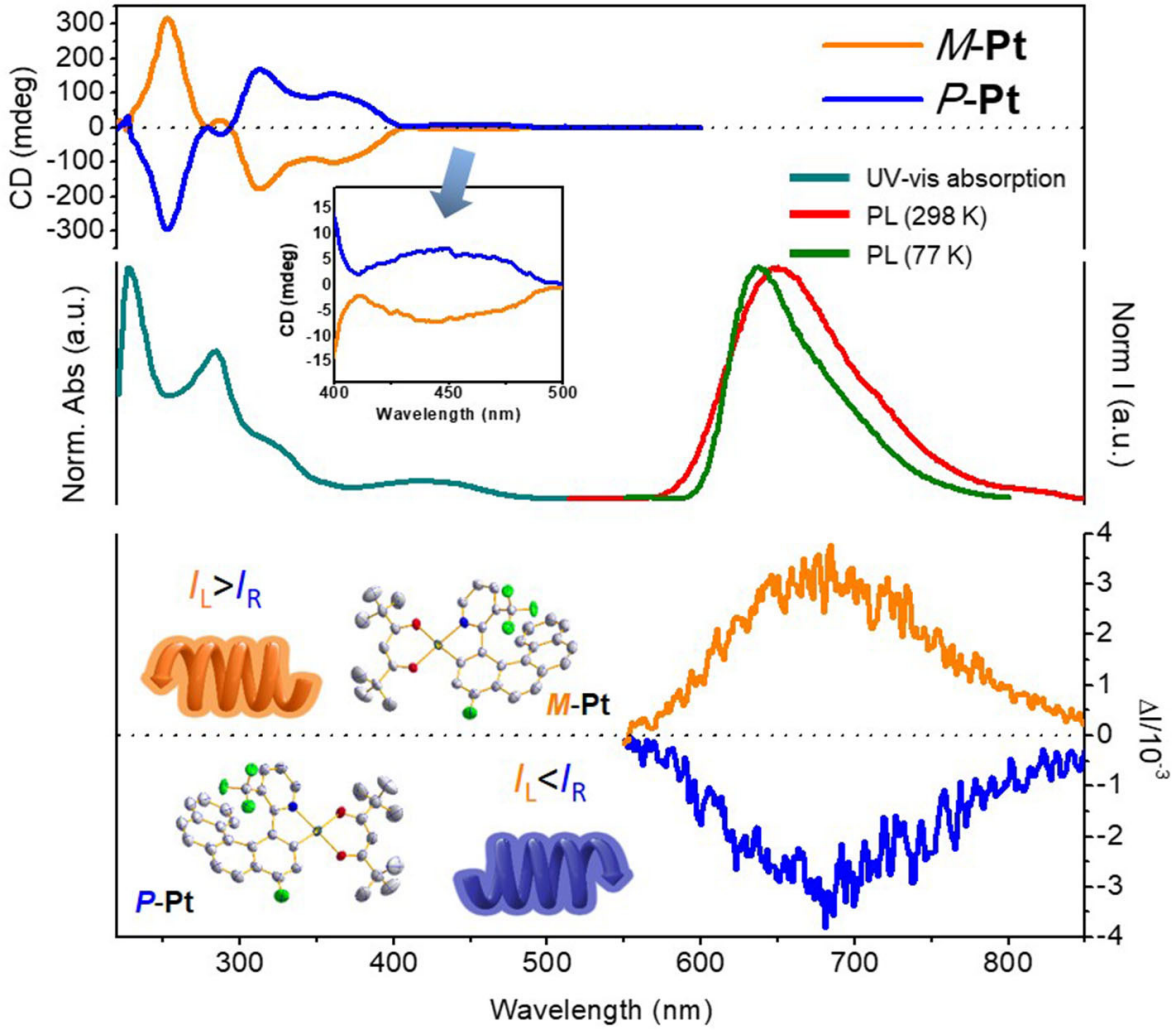
Photophysical properties of platinahelicene in CH_2_Cl_2_ (5 × 10^−5^ mol L^−1^): (top) CD spectra of *P*-**Pt** and *M*-**Pt**; (middle) Normalized absorption and PL spectra of (*RAC*)-**Pt**; (bottom) CPL spectra of *P*-**Pt** and *M*-**Pt**.

To clarify the photophysical properties of (*RAC*)-**Pt**, room temperature UV-vis absorption spectrum in CH_2_Cl_2_ is depicted in Figure 3 (middle). The short-wavelength absorption bands at 227 and 284 nm are mainly associated with π-π^*^ transition, while the weak absorption bands peaking at 350–500 would be attributed to the mixed singlet and triplet metal-to-ligand charge transfer (^1^MLCT and ^3^MLCT) transitions because of the strong spin-orbital coupling effect of the Pt atom. The room temperature PL spectrum of (*RAC*)-**Pt** was further studied with the emission peak at 650 nm and the Commission Internationale de L'Eclairage (CIE) coordinates of (0.68, 0.32) demonstrating the deep-red emission. When measured at 77 K, the emission peak has a blue shift of 14 nm with the emission peak at 636 nm.

Furthermore, emission lifetime (τ) and absolute photoluminescence quantum yield (PLQY) were recorded for (*RAC*)-**Pt** in CH_2_Cl_2_ under nitrogen atmosphere. The phosphorescence lifetime of (*RAC*)-**Pt** (τ = 4.6 μs) is in range of the similar reported Pt(II) complexes (Shen et al., 2014). Moreover, as shown in Figure S9, the linear fit curve on logarithmic scale indicates the phosphorescent emission is basically the only pathway. But due to the deep-red emission with tail to the near-infrared region, the PLQY of (*RAC*)-**Pt** is only about 4%.

### Chiroptical Property

CD spectra were reported for both enantiomers *P*-**Pt** and *M*-**Pt** in CH_2_Cl_2_. As depicted in Figure 3 (top), *P*-**Pt** shows a strong negative cotton effect at 249 nm and positive cotton effects at 313, 363 and 446 nm, as well as corresponding to the UV-Vis absorption spectrum. The *M*-**Pt** reveals exactly the opposite CD spectrum further indicating that the two components are a pair of enantiomers. Furthermore, in the range of 350–500 nm, relatively weak cotton effect is observed, indicating the circular dichroism of the MLCT process.

CPL spectra of *P*-**Pt** and *M*-**Pt** were also preformed [Figure 3 (bottom)] to prove the chiroptical properties of the luminescent molecular upon excitations. The *P*-**Pt** shows negative CPL signals in 550–850 nm region while the *M*-**Pt** shows symmetrical signals in the same position with the |*g*_PL_| of about 6.0 × 10^−3^ which is superior compared with the reported chiral Pt(II) complexes (Shen et al., 2014; Fu et al., 2019). In order to explore the application of materials in OLEDs, the CPL properties of *P*-**Pt** and *M*-**Pt** in doped films were also investigated. When the enantiomers were doped into the host material 26DCzPPy (2,6-bis(3-(9H-carbazol-9-yl)phenyl)pyridine) at a concentration of 5 wt% by vacuum evaporation, the |*g*_PL_| factors are smaller than that in the solution state with the values about 4.0 × 10^−3^. Apparently, as for the CPL performance of *P*/*M*-**Pt**, the host material 26DCzPPy has a certain weakening effect. However, in order to obtain better device performance, 26DCzPPy was selected to further fabricate OLED owing to its unique bipolar characteristic.

### CP-OLED Characterization

**Figure 4:**
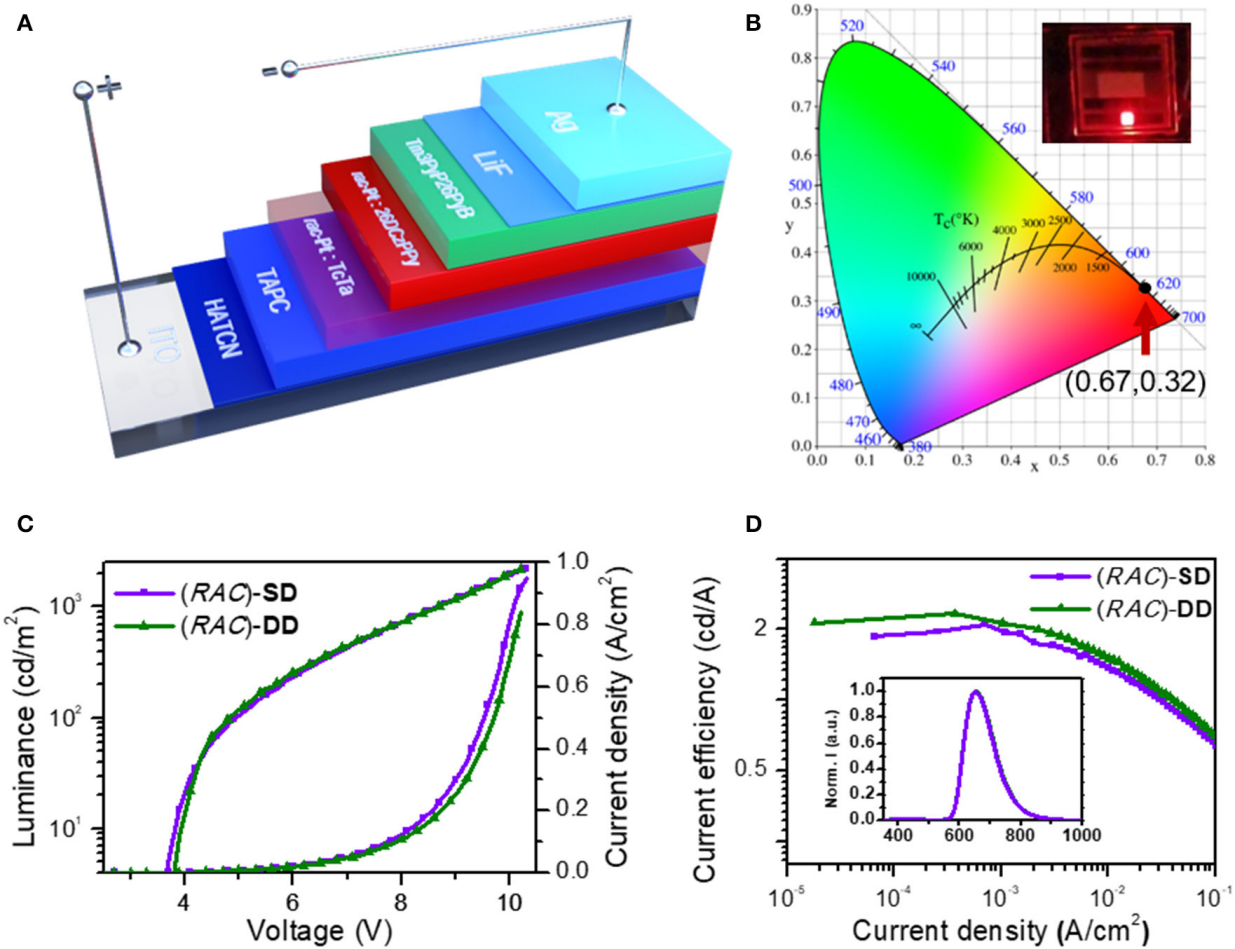
Characteristics of (*RAC*)-**SD** (without TcTa layer) and (*RAC*)-**DD** (with TcTa layer): **(A)** device configuration of OLEDs; **(B)** CIE (x,y) coordinates of both devices; **(C)** current density-luminance-voltage (*J*-*L*-V) curves; **(D)** current efficiency-current density curves (inset: the normalized EL spectrum at 8 V).

Inspired by the unique phosphorescent emission, good thermal and configurational stability and chiroptical property, the application of these platinahelicene in the circularly polarized electroluminescent devices were also investigated. Considering the similar properties of enantiomers and racemate, initially (*RAC*)-**Pt**, was chosen to optimize the device structures. After reasonable structural optimization, the device adopting the single emissive layer of ITO/ HATCN (hexaazatriphenylene-hexacarbonitrile, 6 nm)/HATCN (0.2 wt%): TAPC (di-(4-(*N, N*-ditolyl-amino)phenyl)cyclohexane, 50 nm)/(*RAC*)-**Pt** (5 wt%): 26DCzPPy (10 nm)/Tm3PyP26PyB (1,3,5-*tris*(6-(3-(pyridin-3-yl)phenyl)pyridin-2-yl)benzene, 60 nm)/LiF (1 nm)/Al (100 nm) named (*RAC*)-**SD** showed the best performances (Figure 4A, Figure S13). Respectively, HATCN and LiF are served as the hole-injection and the electron-injection layers; TAPC and Tm3PyP26PyB are used as hole-transporting and electron-transporting layers; Bipolar 26DCzPPy is used as host material which could transport both hole and electrons. Meanwhile, trace HATCN co-doped with TAPC could reduce hole-injection potential energy to achieve low turn-on voltage. The EL spectra, luminance-voltage-current density (*L*-*V*-*J*) and current efficiency-current density characteristics of (*RAC*)-**SD** are depicted in Figure 4, and the key device data are summarized in Table 1. The device (*RAC*)-**SD** displays deep-red EL emission with the peak at 653 nm and the CIEs of (0.67, 0.32) are attained. Moreover, the turn-on voltage of (*RAC*)-**SD** is 3.6 V with the *L*_max_, η_c, max_, maximum external quantum efficiency (EQE_max_) and maximum power efficiency (η_p, max_) of 2,222 cd/m^2^, 2.29 cd/A, 4.16% and 1.90 lm/W, respectively. Furthermore, in order to obtain better device performances, the double emissive layers device of ITO/ HATCN/ HATCN (0.2 wt%): TAPC (50 nm)/(*RAC*)-**Pt** (5 wt%): TcTa (4,4′,4″-tris(carbazol-9-yl)triphenylamine, 10 nm)/ (*RAC*)-**Pt** (5 wt%): 26DCzPPy (10 nm)/Tm3PyP26PyB (60 nm)/LiF (1 nm)/Al (100 nm) named (*RAC*)-**DD** was also fabricated. Generally, the introduction of TcTa to fabricate double emissive layers device could broaden electron-hole recombination area which would enhance the use of electron-hole pairs. As a result, there is a certain improvement of the device performances with the *L*_max_ of 2,201 cd/m^2^, η_c, max_ of 2.33 cd/A, EQE_max_ of 4.44% and η_p, max_ of 1.88 lm/W, respectively.

**Table 1:** EL performances of the devices based on (*RAC*)-**Pt**, *P*-**Pt**, and *M*-**Pt**.

**Device**	**V_**on**_^a^**	***L*_**max**_^b^**	**η_**c, max**_^c^**	**η_**c, *L*1000**_^d^**	**EQE_**max**_^e^**	**η_**p, max**_^f^**	***g*_**EL**_^g^**
	**[V]**	**[cd/m2]**	**[cd/A]**	**[cd/A]**	**[%]**	**[lm/W]**	**[10^**−3**^]**
(*RAC*)-**SD**	3.6	2,222	2.29	0.47	4.16	1.90	–
(*RAC*)-**DD**	3.6	2,201	2.33	0.52	4.44	1.88	–
*P*-**SD**	3.4	1,927	2.26	0.47	4.42	2.03	−1.1
*M*-**SD**	3.4	2,156	2.14	0.36	4.17	1.91	1.1
*P*-**DD**	3.6	1,754	2.23	0.47	4.06	1.87	−0.9
*M*-**DD**	3.6	1,840	2.32	0.48	4.60	1.97	1.3

a Turn-on voltage recorded at a luminance of 1 cd/m^2^;

b Maximum luminance;

c Maximum current efficiency;

d Current efficiency at 1,000 cd/m^2^;

e Maximum external quantum efficiency;

f Maximum power efficiency;

g *g_EL_ around maximum emission peak*.

**Figure 5:**
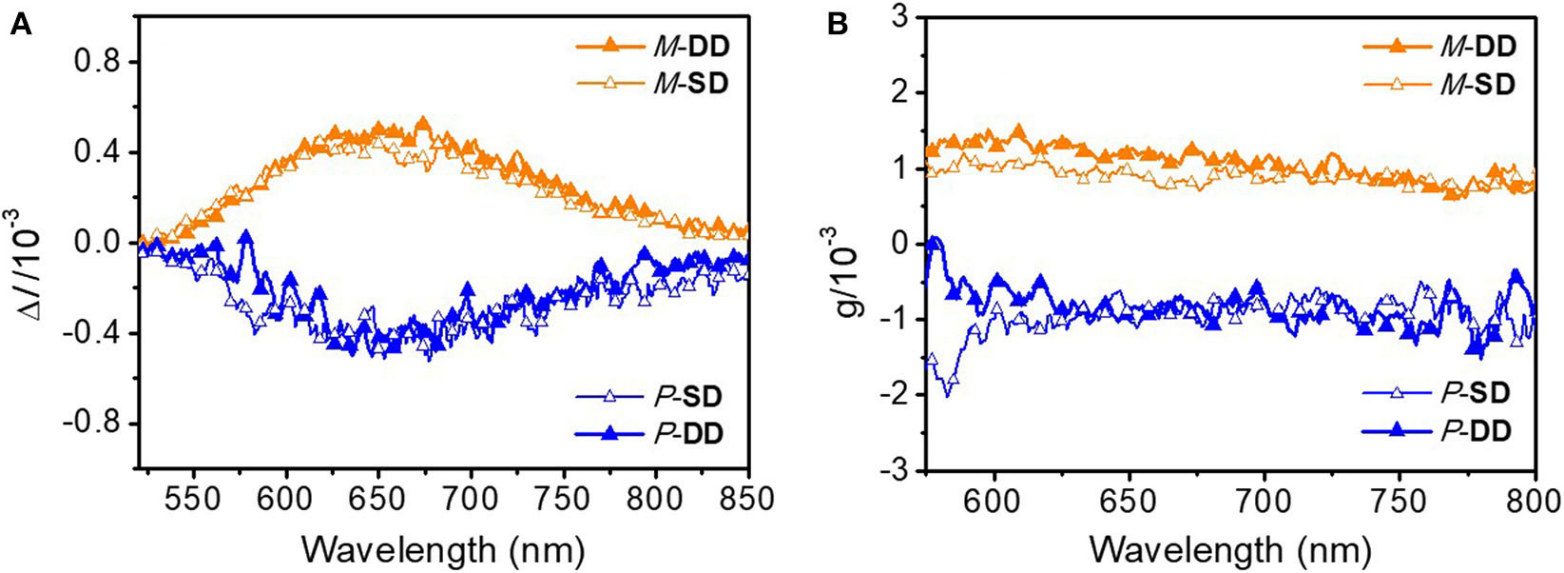
**(A)** CPEL spectra of *P*-**SD**, *M*-**SD**, *P*-**DD**, and *M*-**DD** based on Δ*I* and **(B)** *g*_EL_ vs. wavelength curves of *P*-**SD**, *M*-**SD**, *P*-**DD**, and *M*-**DD**.

Notably, as shown in Figure 5, the two different enantiomer-based OLEDs show obvious CPEL properties with opposite *g*_EL_ of −1 × 10^−3^ and 1 × 10^−3^ at 653 nm for *P*-**Pt** and *M*-**Pt**, respectively, which demonstrate the successful preparation of CP-OLEDs and the device performances of *P*-**SD**, *M*-**SD**, *P*-**DD**, and *M*-**DD** are listed in Table 1 and Figure S13. Notably, although the device performance of *P*-**SD** shows better performance at low current density, *P*-**DD** with wider recombination area performs better at higher current density. However, it is obvious that the measured |*g*_EL_| factors are smaller than the chiral material in solution and film. The reason may be the reflected circularly polarized electroluminescence from metal electrode causing the spiral direction of CP light reverse which impair the intensity of CPEL (Zinna et al., 2017; Yan et al., 2019a). Anyway, successfully obtained CPEL signals indicate a feasible way to fabricate evaporated CP-OLEDs. Meanwhile, appropriate host material and device structure may also be required for better CPEL and EL performances.

## Conclusion

In summary, a pair of chiral platinahelicene enantiomers with excellent thermal and configurational stability was designed and synthesized. Meanwhile, owing to the special structure design strategy, *P*-**Pt** and *M*-**Pt** show good CPL properties possessing |*g*_PL_| of 6.0 × 10^−3^ in solution and 4.0 × 10^−3^ in doped film, respectively. And the fabricated devices *P*-**SD**, *M*-**SD**, *P*-**DD**, and *M*-**DD** exhibited deep-red emission with the emission peak around 653 nm and the EQE_max_ of 4.6% as well as the |*g*_EL_| on the order of 10^−3^.

## Data Availability Statement

All datasets generated for this study are included in the article/Supplementary Material.

## Author Contributions

Z-PY designed and synthesized the platiahelicene complexes, carried out relevant measurement, and wrote the manuscript. X-FL helped in material synthesis and purification. KL helped in the theoretical calculation. Y-XZ and J-LZ designed the whole research.

## Conflict of Interest

The authors declare that the research was conducted in the absence of any commercial or financial relationships that could be construed as a potential conflict of interest.
