# Supplementary material for: Rational Design of the Platinahelicene Enantiomers for Deep-Red Circularly Polarized Organic Light-Emitting Diodes
Source: Front Chem. 2020 Jun 17;8:501. doi: 10.3389/fchem.2020.00501 (PMC7311825; doi:10.3389/fchem.2020.00501)
Supplement: Supplementary file 1 [file Data_Sheet_1.docx]

**Supporting Information**

**Rational design of the platinahelicene enantiomers for deep-red circularly polarized organic light-emitting diodes**

Zhi-Ping Yan, Xu-Feng Luo, Kang Liao, You-Xuan Zheng*, Jing-Lin Zuo*

State Key Laboratory of Coordination Chemistry, Jiangsu Key Laboratory of Advanced Organic Materials, School of Chemistry and Chemical Engineering, Nanjing University, Nanjing 210093, P. R. China

*** Correspondence:**Corresponding Author
[yxzheng@nju.edu.cn](mailto:yxzheng@nju.edu.cn), zuojl@nju.edu.cn

**Content**

**S1. High resolution mass spectrum of (*RAC*)-Pt**

**S2. Chiral HPLC analysis of (*RAC*)-Pt enantiomers**

**S3. NMR spectra for *P*-Pt and *M*-Pt**

**S4. X-ray crystallographic data**

**S5. Photophysical data for (*RAC*)*-*Pt, *P-*Pt and *M-*Pt**

**S6 Electrochemical data**

**S7. Thermal stability**

**S8. Device performance characterization of *P*-SD, *M*-SD, *P*-DD and *M*-DD**

**S1. High resolution mass spectrum of (*RAC*)-Pt**


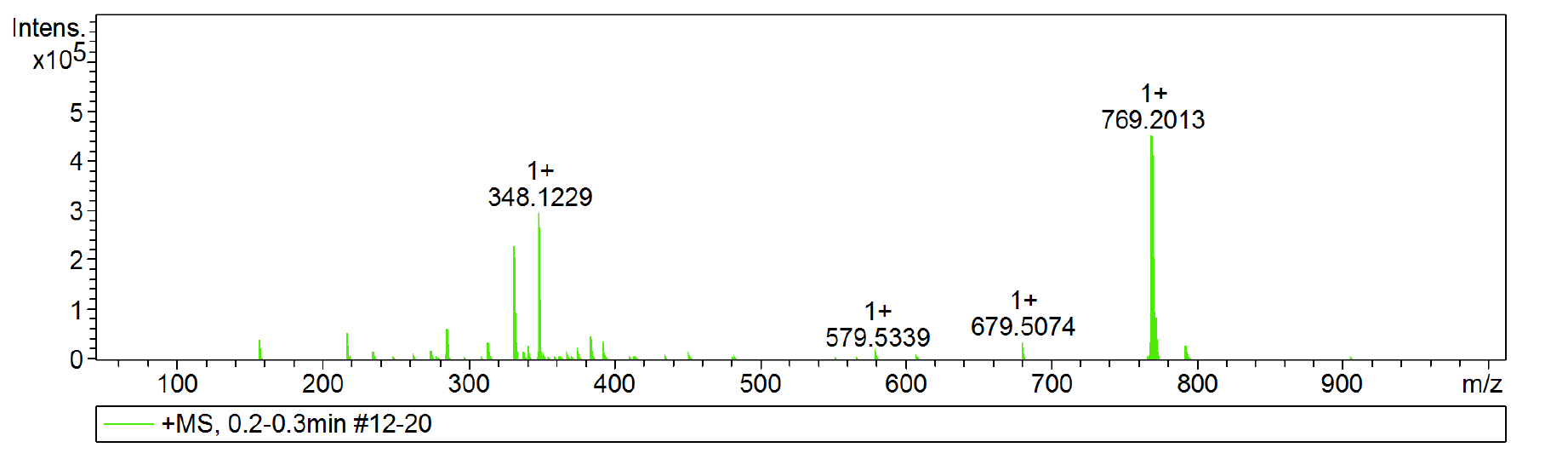


**Figure S1**. High resolution mass spectrum of (*RAC*)-**Pt**.

**S2. Chiral HPLC analysis of (*RAC*)-Pt enantiomers**

**Figure S2**. Chiral HPLC profile of (*RAC*)-**Pt**.

<Column Performance Report>

| Peak# | Ret. Time | Area | Area% | T.Plate# | Tailing F. | Resolution |
| --- | --- | --- | --- | --- | --- | --- |
| 1 | 3.812 | 2470126 | 49.684 | 6377 | 1.268 | -- |
| 2 | 5.976 | 2501570 | 50.316 | 7745 | 1.262 | 9.360 |

**Figure S3**. Chiral HPLC profile of *P*-**Pt**.

<Column Performance Report>

| Peak# | Ret. Time | Area | Area% | T.Plate# | Tailing F. | Resolution |
| --- | --- | --- | --- | --- | --- | --- |
| 1 | 3.726 | 4732240 | 99.892 | 6151 | 1.268 | -- |
| 2 | 6.058 | 5118 | 0.108 | 7182 | 1.977 | 9.802 |

**Figure S4**. Chiral HPLC profile of *M*-**Pt**.

<Column Performance Report>

| Peak# | Ret. Time | Area | Area% | T.Plate# | Tailing F. | Resolution |
| --- | --- | --- | --- | --- | --- | --- |
| 1 | 3.776 | 4019 | 0.110 | 7134 | 1.021 | -- |
| 2 | 5.827 | 3639025 | 99.890 | 7363 | 1.314 | 9.108 |

**S3. NMR spectra for *P*-Pt and *M*-Pt**


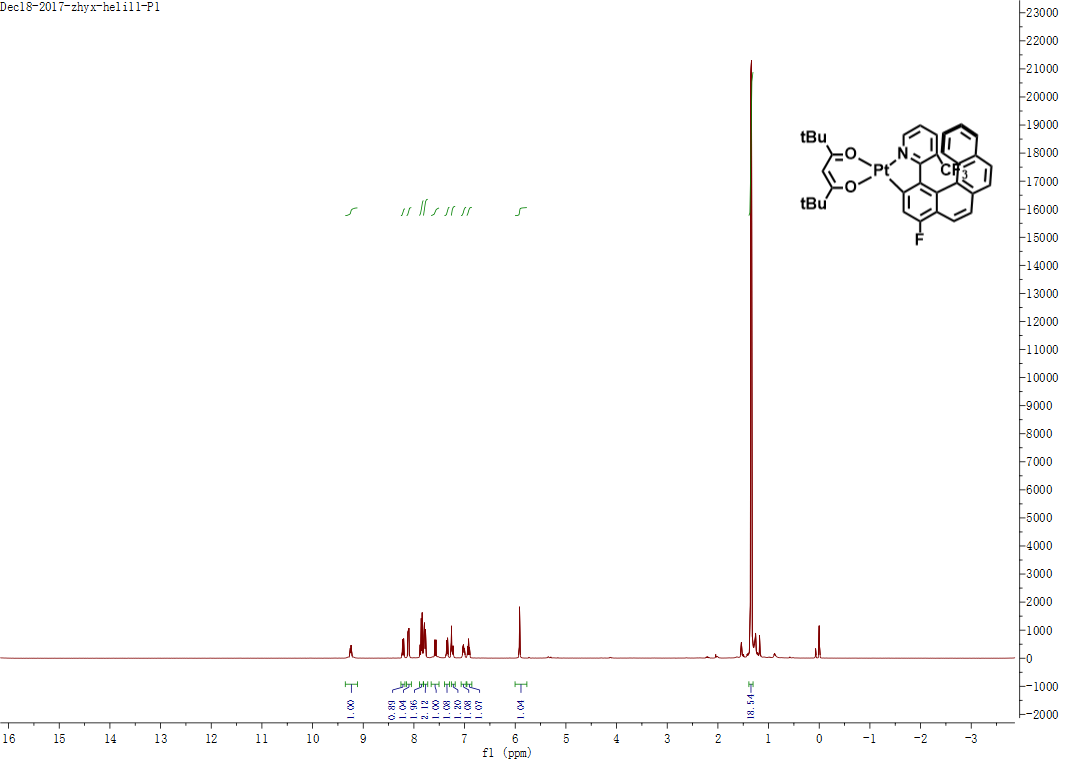


**Figure S5**. ^1^H NMR spectrum of *P*-**Pt**.


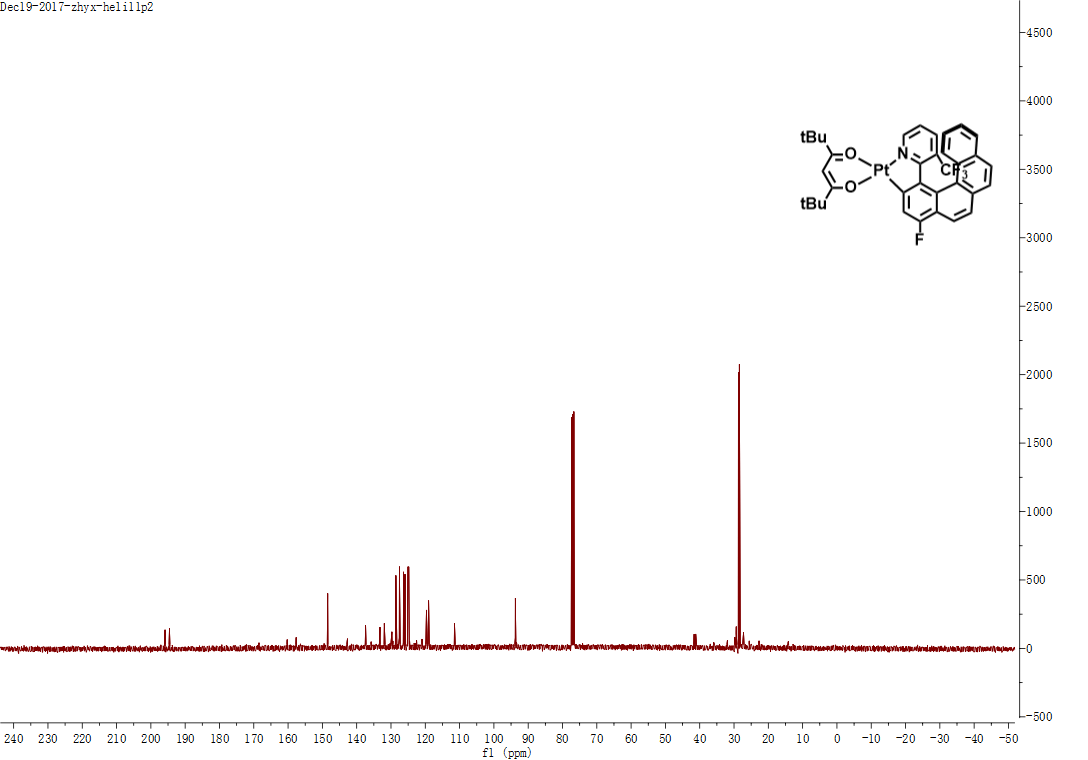


**Figure S6**. ^13^C NMR spectrum of *P*-**Pt**.


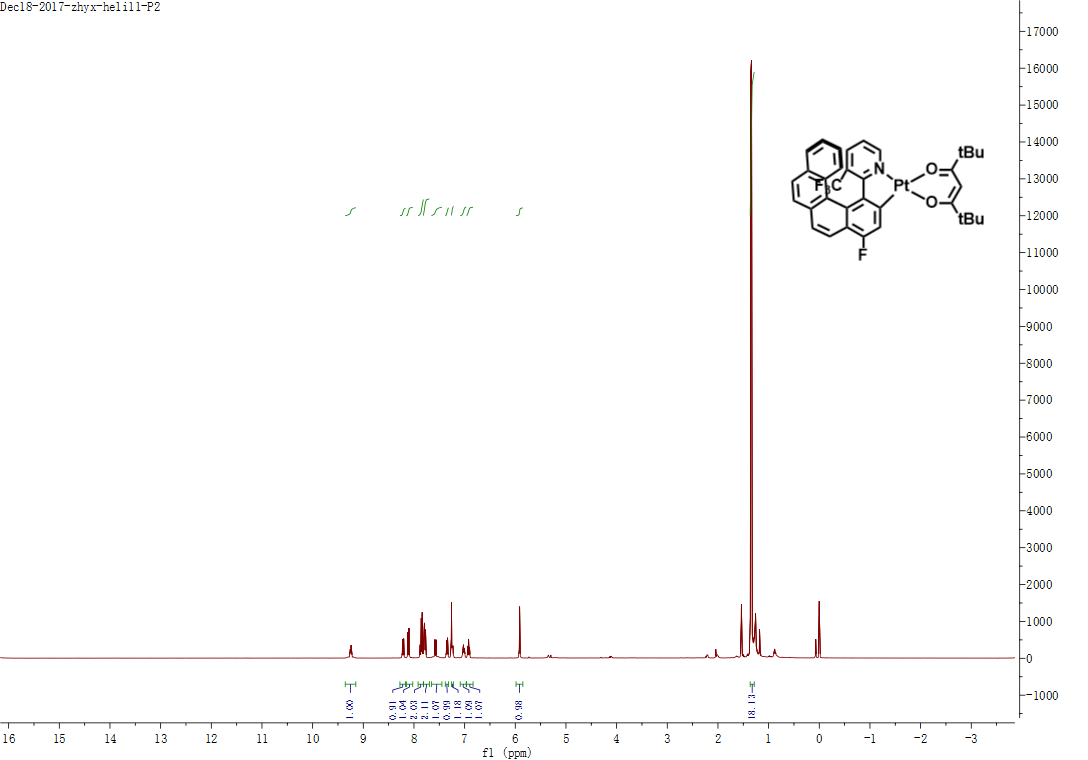


**Figure S7**. ^1^H NMR spectrum of *M*-**Pt**.


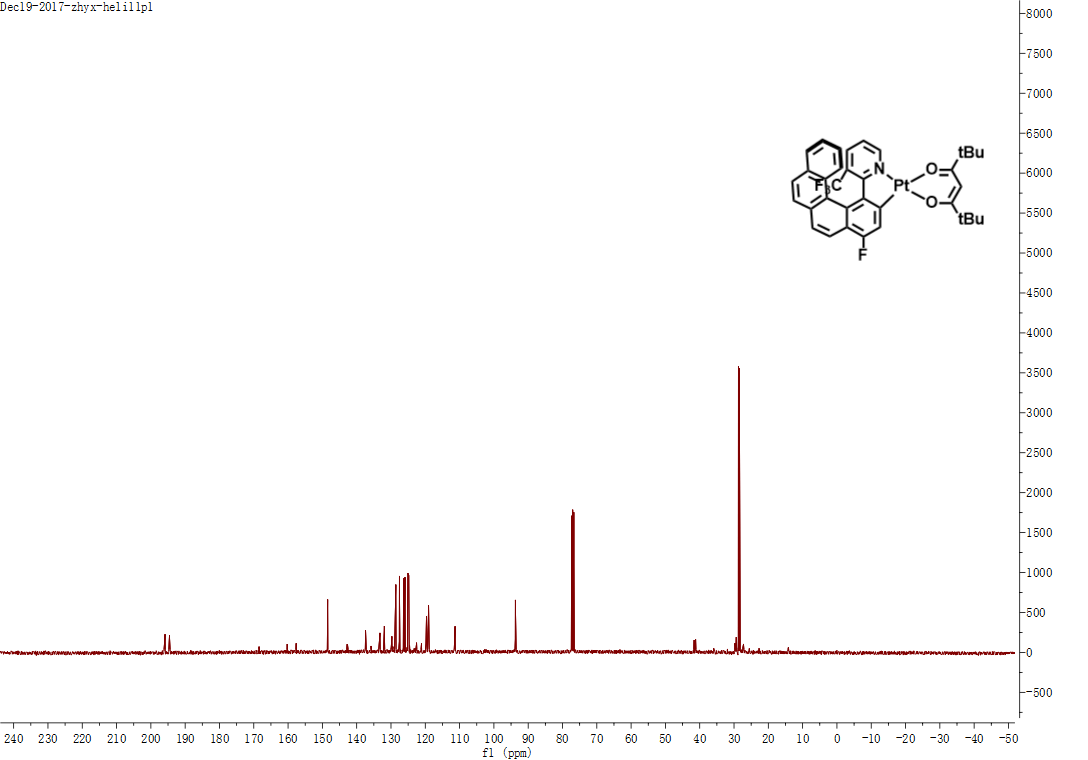


**Figure S8**. ^13^C NMR spectrum of *M*-**Pt**.

**S4. X-ray crystallographic data**

**Table S1**. Crystal data and structure refinement for *P*-**Pt** and *M*-**Pt**.

| Identification code | *P*-**Pt** | *M*-**Pt** |
| --- | --- | --- |
| CCDC | 1844425 | 1844426 |
| Empirical formula | C_35_H_31_F_4_NO_2_Pt | C_35_H_31_F_4_NO_2_Pt |
| Formula weight | 768.7 | 768.7 |
| Temperature/K | 296(2) | 296(2) |
| Crystal system | Orthorhombic | Orthorhombic |
| Space group | P2_1_2_1_2_1_ | P2_1_2_1_2_1_ |
| a/Å | 10.3804(11) | 10.3788(9) |
| b/Å | 12.2412(13) | 12.2284(10) |
| c/Å | 24.215(2) | 24.193(2) |
| α/° | 90 | 90 |
| β/° | 90 | 90 |
| γ/° | 90 | 90 |
| Volume/Å^3^ | 3077.0(6) | 3070.5(4) |
| Z | 4 | 4 |
| ρ_calc_g/cm^3^ | 1.659 | 1.663 |
| μ/mm^‑1^ | 4.616 | 4.626 |
| F(000) | 1512 | 1512 |
| Radiation | MoKα (λ = 0.71073) | MoKα (λ = 0.71073) |
| 2Θ range for data collection/° | 3.36 to 54.46 | 3.36 to 55.02 |
| Index ranges | -13 ≤ h ≤ 13, -15 ≤ k ≤ 9, -30 ≤ l ≤ 31 | -13 ≤ h ≤ 13, -15 ≤ k ≤ 11, -31 ≤ l ≤ 31 |
| Reflections collected | 20528 | 20924 |
| Independent reflections | 6852 [R_int_ = 0.0369, R_sigma_ = 0.0545] | 7050 [R_int_ = 0.0436, R_sigma_ = 0.0608] |
| Data/restraints/parameters | 6852/890/394 | 7050/896/394 |
| Goodness-of-fit on F^2^ | 1.117 | 0.996 |
| Final R indexes [I>=2σ (I)] | R_1_ = 0.0313, wR_2_ = 0.0618 | R_1_ = 0.0337, wR_2_ = 0.0753 |
| Final R indexes [all data] | R_1_ = 0.0377, wR_2_ = 0.0633 | R_1_ = 0.0425, wR_2_ = 0.0786 |
| Largest diff. peak/hole / e Å^-3^ | 0.70/-0.64 | 0.76/-0.81 |
| Flack parameter | 0.007(8) | 0.009(9) |

*R*_1_^a^ = Σ||*F*_o_| − |*F*_c_||/Σ*F*_o_|. wR_2_^b^ = [Σ*w*(*F*_o_^2^ − *F*_c_^2^)^2^/Σ*w*(*F*_o_^2^)]^1/2^

**S5. Photophysical data for (*RAC*)*-*Pt, *P-*Pt and *M-*Pt**

**Figure S9**. Transient decay curve of (*RAC*)-**Pt** in CH_2_Cl_2_ (5×10^-5^ M).

**Figure S10**. *g*_PL_ vs. wavelength curves of *P*-**Pt** and *M*-**Pt** in (a) CH_2_Cl_2_ (5×10^-5^ M) and (b) doped film (5 wt% in 26DCzPPy).

**Figure S11**. CD spectra of *P*-**Pt** and *M*-**Pt** before (dash) and after sublimation (solid).

**Table S2**. Photophysical data for (*RAC*)-**Pt**.

| Complex Configuration | | λ_PL_ nm | λ_ECD_ nm (mdeg) | *g*_PL_ /10^-3^ | | *τ* μs | Ф |
| --- | --- | --- | --- | --- | --- | --- | --- |
|  |  |  |  | Solution | film |  |  |
| (*RAC*)-**Pt** | *P* | 650 | 253(-277),287(-15),314(162),361(95),446(7) | -5.9 | -3.8 | 4.6 | 0.04 |
|  | *M* |  | 253(296),287(16),314(-172),361(-100),446(-7) | 6.0 | 4.2 |  |  |

**S6 Electrochemical data**

**Table S3**. Electrochemical data of *P*-**Pt** and *M*-**Pt**.

| Complex | *E*_ox,onset (Pt)_^a^ V | *E*_red,onset (Pt)_^b^ V | *E*_ox,onset (Fe)_^c^ V | *E_g,_*_opt_^d^ eV | *E*_HOMO_/*E*_LUMO_^e^ eV |
| --- | --- | --- | --- | --- | --- |
| *P*-**Pt** | 0.83 | -1.17 | 0.05 | 2.34 | -5.58/ -3.24 |
| *M*-**Pt** | 0.83 | -1.19 | 0.05 | 2.34 | -5.58/ -3.24 |
| ^a^: The onset of oxidation of Pt(II) complexes; ^b^: The onset of reduction curves of Pt(II) complexes; ^c^: The onset of oxidation curves of Ferrocene; ^d^: Optical gap (= 1240/λ_onset_); ^e^: *E*_HOMO_ = -[*E*_ox_ - *E*_(Fc/Fc+)_ + 4.8] eV, *E*_LUMO_ = *E*_HOMO_ + *E*_g_. | | | | | |

**S7. Thermal stability**

**Figure S12**. TGA curve of (*RAC*)-**Pt** recorded under nitrogen at a heating rate of 10 ^o^C min^-1^.

**S8. Device performance characterization of *P*-SD, *M*-SD, *P*-DD and *M*-DD**

**Figure S13.** (a) Device configurations of CP-OLEDs; (b) Chemical structures of adopted materials.

**S12. Device performance characterization of *P*-SD, *M*-SD, *P*-DD and *M*-DD**

**Figure S14.** Characteristics of devices *P*-**SD**, *M*-**SD**, *P*-**DD** and *M*-**DD**: (a) Current density-luminance-voltage (*J*-*L*-V) curves; (b) Current efficiency-current density curves (inset: normalized EL spectra at 8 V).
